# Comparative effects of dry-aging and wet-aging on physicochemical properties and digestibility of Hanwoo beef

**DOI:** 10.5713/ajas.19.0031

**Published:** 2019-08-03

**Authors:** Ji-Han Kim, Tae-Kyung Kim, Dong-Min Shin, Hyun-Wook Kim, Young-Boong Kim, Yun-Sang Choi

**Affiliations:** 1 Department of Food Science and Biotechnology of Animal Resources, Konkuk University, Seoul 05029, Korea; 2 Research Group of Food Processing, Korean Food Research Institute, Wanju 55365, Korea; 3 Department of Animal Science and Biotechnology, Gyeongnam National University of Science and Technology, Jinju 52725, Korea

**Keywords:** Wet Aging, Dry Aging, Digestibility, Shear Force, Beef Loin

## Abstract

**Objective:**

The purpose of this study was to investigate the effects of aging methods (AM) i.e. dry-aging (DA) and wet-aging (WA) on the physicochemical properties and *in vitro* digestibility of proteins in beef short loin.

**Methods:**

Short loins (*M. longissmus lumborum*), were trimmed and boned-out on the fifth day postmortem, from a total of 18 Hanwoo, which were purchased from a commercial slaughterhouse. Short loins were separated randomly grouped into one of the three treatments: control, WA (1°C, 7 days), and DA (1°C, 0.5 m/s, 85% relative humidity [RH], 30 days).

**Results:**

Dry-aged beef (DAB) exhibited higher pH, water holding capacity (WHC), myofibrillar fragmentation index (MFI), and digestibility, however lower lightness, redness, and yellowness values, cooking loss, and shear force (SF), than those of wet-aged beef (WAB) (p<0.05). The myosin light chain band intensity of DAB was higher than that of control and WAB in sodium dodecyl sulfate-polyacrylamide gel electrophoresis. The *in vitro* digestibility of aged beef was highly (p<0.001) correlated to physicochemical properties except WHC. The correlation coefficient between AMs and WHC was higher than that between AM and SF (p<0.05) or MFI (p<0.001). A high correlation was observed between SF and MFI (p<0.001).

**Conclusion:**

Thus, we believe that DAB is more advantageous than WAB owing to its high digestibility and WHC and low SF.

## INTRODUCTION

Meat quality is a very important aspect to the consumers and the meat industry. In fact, the most important factors of beef quality are tenderness and juiciness. Therefore, in accordance with the consumer preferences, a high-marbling attribute has a positive impact on the palatability of beef [1]. However, to be preferred, the low-marbled beef needs to be improved in various ways to improve its poor taste, toughness, and dryness. Various efforts are being taken up by mainly using mechanical methods to improve the palatability of low-marbled beef [2].

Aging is another approach used to improve the tenderness, flavor, and juiciness i.e. water holding capacity (WHC) of beef and is recently being used to improve low-marbled products. Yim et al [3] reported that aging method (AM) is being widely used to improve the meat tenderness and juiciness, by causing complex changes in the muscle metabolism. The AM is utilized to enhance the tenderness and aid in flavor development of beef products. Aging is normally processed through either wet-aging (WA) or dry-aging (DA) [4]. The WA is more commonly used and refers to allowing meat to age by storing it in a sealed barrier package such as vacuum package at refrigerated temperatures for up to 7 days [5]. The DA is an expensive procedure that needs a lot of space and time. It causes a significant level of shrinkage and generates a high amount of crust with excessively dried wastes that must be trimmed [1]. The dry-aged beef (DAB) had higher trim loss than wet-aged beef (WAB) [6]. Moreover, the storage of DAB is relatively expensive compared to WAB because of the shrinkage and wastage of beef directly related to the DAB procedure [7]. Smith et al [5] reported that DAB is a successful process and is used at special outlets to meet the needs of consumers who prefer this unique beef flavor. A few researchers have studied the variations in the characteristics of WAB and DAB with respect to their quality [1,5–8]. Furthermore, studies were conducted on the quality variations between WA and DA processes [4,6]. However, regarding digestibility, the comparison between WAB and DAB and correlation studies remain unexplored.

Therefore, the objectives of this study were to understand the influence of AM on the digestibility and correlation between the type of AM and physicochemical properties of beef.

## MATERIALS AND METHODS

### Samples preparation and aging methods

Short loins (*M. longissmus lumborum*), were trimmed and boned-out on the 5 days postmortem, from a total of 18 Hanwoo (quality grade 1, approximately 26 month age), which were purchased from a commercial slaughterhouse. Short loins were separated randomly grouped into one of the three treatments: control, WA (1°C, 7 days), and DA (1°C, 0.5 m/s, 85% relative humidity [RH], 30 days) [9]. Each side was equally represented among the aging treatments. Each loin section was relocated within the individual testing chambers on weekly basis to avoid any location effect on meat quality attributes. Each short loin designated for WA was weighed in a vacuum package and placed in cooler on a stainless-steel rack. The Hanwoo loin sections were removed from the assigned aging test chamber. The loss in weight and time taken for surface trimming were recorded. Steak samples were cut from each loin section to assess the meat physicochemical properties and perform digestibility analyses. All the processes were performed in triplicates.

### Cooking methods

Samples were placed at room temperature (20°C) for 30 min prior to cooking and cut into cubes (3×3×2.54 cm, diameter× diameter×thickness). The samples were cooked at 145°C using preheated electronic grills. The core temperature of samples reached 71.1°C, as measured using a digital thermocouple machine (Tes-1305, Tes Electrical Co., Taipei, Taiwan) equipped with a data logger (RS-232, Tes Electrical Co., Taiwan).

### Experiment methods

The samples (5 g) were added with 20 mL of distilled water and homogenized. Then, pH was measured using a pH meter (Model 340, Mettler-Toledo GmbH, Schwerzenbach, Switzerland) calibrated with pH 4.0, 7.0, and 10.0 solutions. The color properties of samples were determined using a colorimeter (Minolta Chroma meter CR-210, Minolta Ltd., Osaka, Japan, calibrated with a white plate, Commission Internationale de l’Eclairage (CIE) L* value: 97.83, CIE a* value: 0.43, CIE b* value: 1.98, 2° observer, D65) after blooming for 30 min. We measured the initial sample weight, weight after cooking was measured after the cooked samples were cooled to room temperature (21°C) for 3 h and the cooking weight loss was deduced. The WHC was measured using filter paper press as described by Kim et al [10]. After cooking, the six cores per each sample were collected parallel to the muscle fiber shape by using a 1.27 cm diameter handed coring device, and the shear force (SF) was measured using a TA-XT2*i* (Stable Micro System, Scarsdale, NY, USA) equipped with a triangle angular slot cutting edge under a crosshead speed of 1.5 mm/s. Myofibrillar fragmentation index (MFI) was measured using the method described by Culler et al [11]. Sample protein was extracted using MFI buffer solution (20 mM potassium phosphate at pH 7.0 with 100 mM KCl, 1 mM ethylenediaminetetraacetic acid, 1 mM NaN_3_, and 1 mM MgCl_2_). The absorbance of the extract (0.5 mg protein/mL) was evaluated at 540 nm by using a spectrophotometer (Optizen 2120UV, Mecasys, Seoul, Korea). The protein degradation and denaturation in meat was evaluated by using the protein extracts (0.5 mg protein/mL) from the MFI and 12% sodium dodecyl sulfate-polyacrylamide gel electrophoresis (SDS-PAGE) according to the method described by Kim et al [12]. *In vitro* gastrointestinal digestion was conducted using specific steps with 4 phases (mouth, gastric, duodenal, and bile juices) as described by Lee et al [13]. The protein digestibility in the cooked beef during simulated human gastrointestinal digestion was measured by the infiltration rates of dialysis tubing and expressed as the percentage of protein concentration inside and outside the dialysis tubing. The protein concentration was measured using Biuret method.

### Statistical analysis

Six muscles per treatment and AMs (control, WA, and DA) were used as experimental units. The complete assessment was performed in triplicates and significant differences among the replicates (p<0.05) were not observed. The AM was carried as fixed term and replicators and loins were carried as random term. The results were presented as mean values and standard error values in tables. All the statistical analyses were analyzed as completely randomized designs using SPSS Ver. 20.0 (SPSS Inc., Chicago, IL, USA). One-way analysis of variance and Duncan’s multiple testes were used to determine the significant differences among treatments (p<0.05). Correlation coefficients between the variables such as digestibility, pH, L*-value, a*-value, b*-value, cooking weight loss, WHC, SF, and MFI were generated using Pearson’s correlation coefficient option (SPSS Inc., Chicago, IL, USA).

## RESULTS AND DISCUSSION

### Physicochemical properties and *in vitro* digestibility

In beef loins, the effect of AMs on physicochemical properties and *in vitro* digestibility are shown in the Table 1. The pH of control group was lower than that of groups that underwent aging treatments (p<0.05). The pH of DAB was the highest (p<0.05) among the three groups. Aksu et al [14] reported that the increase in pH during aging process is because of the formation of nitrogen compounds from proteolysis. In agreement with the results reported by Kim et al [6] we observed that the pH of DAB was higher than that of WAB. The lightness, redness, and yellowness values of DAB were lower than those of WAB (p<0.05), owing to the high pH and WHC of DAB [6]. In terms of consumer perceptions, higher redness of meat contributes to the increase in meat colour acceptability. In addition, higher lightness (about 35) demonstrated the positive relation with the consumer colour acceptability [15]. These color changes in aged meat could be negatively attributed to the consumer acceptability. The cooking loss of DAB was lower than that of WAB (p<0.05). On the contrary, the WHC of DAB was higher than that of WAB (p<0.05). The decreased cooking loss and increased WHC of DAB might be because of low moisture content caused by evaporation during DA [16]. The decreased SF of DAB compared to that of WAB is due to the high proteolysis index [1]. The MFI and protein digestibility of DAB were higher than that of WAB (p<0.05) owing to the high proteolysis. The aging of meat increased the quantity of small peptides in beef after digestion and the consumption of this meat lowers the blood pressure and causes antioxidant effects [17]. The results of proximate composition from the control, WA and DA were shown as moisture (61.80%±1.13%, 58.94%±1.39%, and 56.73%±1.48%), protein (34.68%±1.28%, 35.90%±1.29%, and 34.98%±1.42%) and fat (4.09%±0.13%, 5.28%±0.66%, and 7.42%±0.81%), respectively.

**Figure 1:**
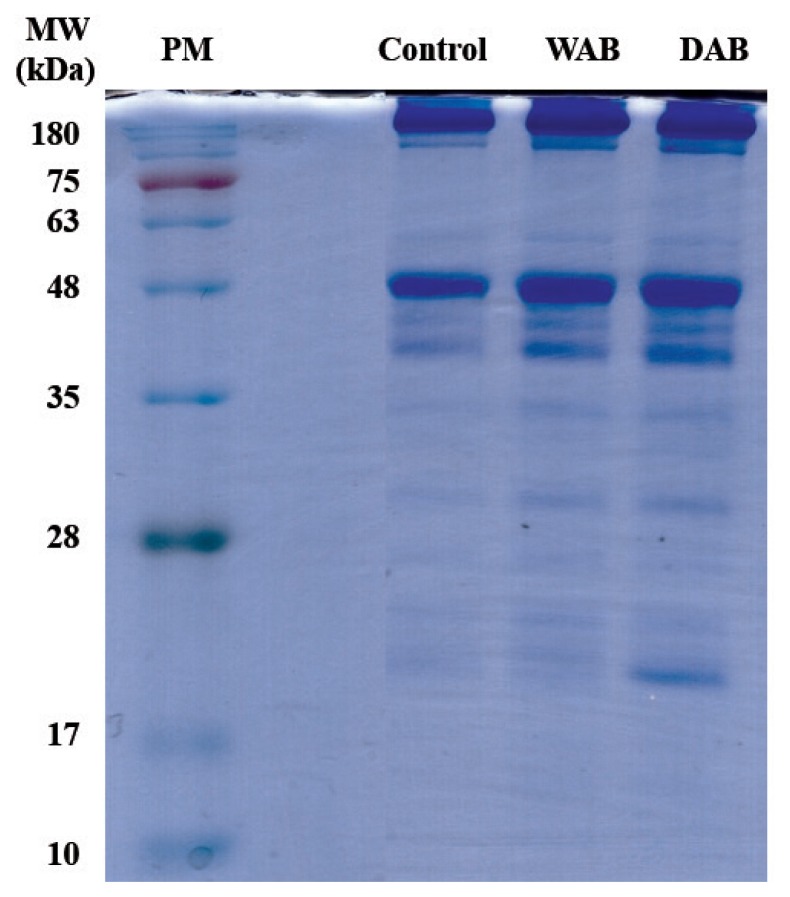
Sodium dodecyl sulfate-polyacrylamide gel electrophoresis patterns of aging methods on beef loin. Vertical numbers indicate molecular weight marker in kDa. PM, protein marker; Control, non-aged beef; WAB, wet-aged beef; DAB, dry-aged beef.

The SDS-PAGE results of protein samples obtained after performing AMs in beef loins is shown in Figure 1. As expected, the aging of beef loins significantly affected the intensity (percentage) of several protein bands (20 to 48 kDa) compared to those of the control group. Toldra [18] reported that the various peptides obtained from protein breakdown were detected during the processing of dry-cured ham. In particular, the myosin light chain band (16 to 27.5 kDa molecular weight) intensity of the DAB protein samples was higher than that of the control and WAB groups. Claeys et al [19] reported that the SDS-PAGE protein separation patterns displayed a range of 3 to 17 kDa proteins in aged beef.

After performing the AMs in beef loins, the Pearson corre lation coefficients among each physicochemical properties according to different AMs (WAB and DAB) are presented in the Table 2. The *in vitro* digestibility of WAB was negatively correlated to pH, a* and SF (p<0.05) whereas the digestibility of DAB showed negatively correlation with SF (p<0.01). The pH of both WAB and DAB was positively correlated with a* and b*. The cooking loss of WAB exhibited a negative correlation with WHC. Therefore, a comparison of various physicochemical properties between AMs i.e. WA and DA might provide the information to improve the digestibility and favorable physicochemical properties.

## CONCLUSION

The objectives of this study were to understand the influence of AM on the digestibility and its correlation with physicochemical properties. The *in vitro* protein digestibility of DAB slightly improved compared to that of WAB. *In vitro* digestibility of aged beef was highly (p<0.001) correlated with all the physicochemical properties except WHC. The correlation coefficient between AMs and WHC was higher than that between AMs and SF (p<0.05) or MFI (p<0.001). The intensity of myosin light chain band (molecular weights from 16 to 27.5 kDa) of DAB was higher than that of control and WAB. Although the contribution of the aging process to the taste of the meat was previously reported, the digestibility of comparing dry and wet aged Hanwoo is probably the first approach. Therefore, it concluded that the DA process affected differences of protein digestibility and degradation to contribute to the improvement of meat quality compared to the WAB.

## Figures and Tables

**Table 1:** Effect aging methods on pH, color, cooking loss, water holding capacity, shear force, myofibrillar fragmentation index, and digestibility (%) of beef loins

Items	Treatments1)		

	Control	WAB	DAB
pH	5.49±0.01^c^	5.53±0.01^b^	5.58±0.01^a^
L*-value	30.48±0.87^a^	28.25±0.87^b^	26.99±0.22^c^
a*-value	17.12±0.26^a^	15.49±0.31^b^	10.84±0.98^c^
b*-value	8.94±0.36^a^	7.41±0.26^b^	5.32±0.63^c^
Cooking loss (%)	35.52±0.14^a^	33.28±0.16^b^	10.62±1.28^c^
WHC (%)	22.62±1.00^b^	23.99±0.46^b^	30.09±0.62^a^
SF (N)	9.78±0.77^a^	8.08±0.35^b^	7.95±0.47^c^
MFI	92.30±2.56^c^	116.63±1.68^b^	173.86±2.34^a^
Digestibility (%)	98.960±0.001^c^	98.972±0.002^b^	98.980±0.001^a^

WHC, water holding capacity; SF, shear force; MFI, myofibrillar fragmentation index.

1) Control, non-aged beef; WAB, wet-aged beef; DAB, dry-aged beef.

a–c Means within a row with different letters are significantly different (p<0.05).

**Table 2:** Pearson correlation coefficients and probabilities of aging methods (WAB, DAB) on pH, color, cooking loss, water holding capacity, shear force, myofibrillar fragmentation index, and digestibility (%) of beef loins

Items	Aging methods	pH	L*-value	a*-value	b*-value	Cooking loss	WHC	SF	MFI
Digestibility	WAB	−0.632*	−0.678	−0.883**	−0.705	−0.037	−0.083	−0.378*	0.478
	DAB	−0.136	−0.334	−0.305	0.265	−0.568	0.122	−0.950**	0.428
pH	WAB	1.000	0.692	0.876*	0.857*	0.105	−0.119	0.555	0.110
	DAB	1.000	−0.515	0.897**	0.877**	0.200	0.352	−0.312	−0.542
L*-value	WAB	-	1.000	0.672	0.874*	0.228	−0.089	0.024	−0.432
	DAB	-	1.000	0.624	0.668	−0.209	−0.012	−0.243	0.569
a*-value	WAB	-	-	1.000	0.756	−0.050	0.078	0.627	−0.205
	DAB	-	-	1.000	0.982**	−0.267	−0.336	0.379	0.392
b*-value	WAB	-	-	-	1.000	0.474	0.394	0.328	−0.259
	DAB	-	-	-	1.000	−0.333	−0.186	0.329	0.525
Cooking loss	WAB	-	-	-	-	1.000	−0.971**	0.167	−0.375
	DAB	-	-	-	-	1.000	−0.692	−0.680	−0.116
WHC	WAB	-	-	-	-	-	1.000	−0.301	0.228
	DAB	-	-	-	-	-	1.000	−0.114	−0.329
SF	WAB	-	-	-	-	-	-	1.000	−0.034
	DAB	-	-	-	-	-	-	1.000	−0.034

WAB, wet-aged beef; DAB, dry-aged beef; WHC, water holding capacity; SF, shear force; MFI, myofibrillar fragmentation index.

** Highly significant statistically at p<0.001.

* Highly significant statistically at p<0.05.
